# UCP2 as a Potential Biomarker for Adjunctive Metabolic Therapies in Tumor Management

**DOI:** 10.3389/fonc.2021.640720

**Published:** 2021-03-08

**Authors:** Frederic A. Vallejo, Steven Vanni, Regina M. Graham

**Affiliations:** ^1^ Department of Neurosurgery, University of Miami Miller School of Medicine, Miami, FL, United States; ^2^ University of Miami Brain Tumor Initiative, Department of Neurosurgery, University of Miami Miller School of Medicine, Miami, FL, United States; ^3^ Sylvester Comprehensive Cancer Center, University of Miami Miller School of Medicine, Miami, FL, United States

**Keywords:** uncoupling protein 2 (UCP2), cancer, glioma, biomarker, metabolism, Warburg effect, Glioblastoma, precision-medicine

## Abstract

Glioblastoma (GBM) remains one of the most lethal primary brain tumors in both adult and pediatric patients. Targeting tumor metabolism has emerged as a promising-targeted therapeutic strategy for GBM and characteristically resistant GBM stem-like cells (GSCs). Neoplastic cells, especially those with high proliferative potential such as GSCs, have been shown to upregulate UCP2 as a cytoprotective mechanism in response to chronic increased reactive oxygen species (ROS) exposure. This upregulation plays a central role in the induction of the highly glycolytic phenotype associated with many tumors. In addition to shifting metabolism away from oxidative phosphorylation, UCP2 has also been implicated in increased mitochondrial Ca^2+^ sequestration, apoptotic evasion, dampened immune response, and chemotherapeutic resistance. A query of the CGGA RNA-seq and the TCGA GBMLGG database demonstrated that UCP2 expression increases with increased WHO tumor-grade and is associated with much poorer prognosis across a cohort of brain tumors. UCP2 expression could potentially serve as a biomarker to stratify patients for adjunctive anti-tumor metabolic therapies, such as glycolytic inhibition alongside current standard of care, particularly in adult and pediatric gliomas. Additionally, because UCP2 correlates with tumor grade, monitoring serum protein levels in the future may allow clinicians a relatively minimally invasive marker to correlate with disease progression. Further investigation of UCP2’s role in metabolic reprogramming is warranted to fully appreciate its clinical translatability and utility.

## Introduction

In recent years, oncologic treatment plans have continued to emphasize the importance of highly specific, personalized, and targeted therapeutic modalities to combat malignancies in the clinic. Whereas some cancers have seen drastic improvements in prognostic outcomes, certain primary brain tumors have proven to be particularly refractory to most drugs. Very little improvement has been made in the management of patients with gliomas and Glioblastomas, specifically, with 5-year survival in adults and children remaining dismally low (at 7.2% and 17.7%, respectively) (1–3). With very few efficacious drugs available to these patients, there exists an obvious and desperate need for novel approaches and new potential interventions to improve patients’ quality of life. Challenges that have historically hindered glioblastoma drug discovery and utility are impermeability of the blood brain barrier, drastic intra-tumoral heterogeneity, being extremely invasive in nature, and the presence of neurocritical structures commonly surrounding the tumor. Isolating and targeting populations of common progenitor cells, or glioma stem-like cells (GSCs), within the tumor has recently emerged as a potentially effective strategy to eliminate the cells responsible for much of the rapid proliferation commonly observed in these pathologies (4, 5).

Neoplastic cells exhibit a multitude of mitotic, biochemical, and cellular aberrancies. Many solid tumors have been shown to exhibit an increased dependency on glycolytic metabolism and may even forgo oxidative phosphorylation in the presence of oxygen. In fact, it is this often-overlooked increased glycolytic flux on the part of cancer cells that enables clinicians to effectively localize tumors and metastatic outgrowths *via* fluorodeoxyglucose positron emission tomography (PET scan) on a regular basis. This phenomenon, originally described by Otto Warburg nearly a century ago, has been highlighted as one of the central tenants of tumorigenesis and an “emerging hallmark of cancer” (6–8). While this switch in metabolism has been well documented in the literature for some time, the underlying mechanism by which malignant cells undergo this transition is still in question.

Existing hypotheses concerning the Warburg effect posit that forgoing oxidative phosphorylation and generating energy exclusively *via* glycolysis may be a more efficient way to generate metabolic intermediates, nucleotides for further proliferation, and drive angiogenesis to the hypoxic, acidic microenvironment. Another theory is that increased competition for shared metabolic resources in the tumor’s vicinity cause the neoplastic cells to seek the path of less resistance and opt for the “evolutionarily less efficient pathway” to generate energy (9). Additionally, this shift in metabolism has been shown to increase cell proliferation as long as the supply of glucose is not limiting. Recent literature suggests that cells may be shutting off mitochondrial cellular respiration by increasing the expression of mitochondrial uncoupling protein 2 (UCP2), in an effort to mitigate the effect of cytotoxic reactive oxygen species (ROS), thereby inducing the Warburg effect *via* UCP2 upregulation (10, 11). In non-cancerous cells, UCP2 upregulation may facilitate, rather than inhibit, continued fatty acid metabolism by mitigating increased ROS generation and actually result in decreased glycolytic flux (12, 13). However, in neurons where basal ROS levels are consistently elevated, as is the case in many cancerous cells, UCP2 upregulation has shown to decrease the cell’s ability to sense glucose appropriately and results in disruption of carbohydrate homeostasis (14, 15).

Rapidly dividing stem cells with high proliferative and anabolic capabilities have been shown to overexpress UCP2, whereas induction of neuronal differentiation causes a loss of UCP2 expression (16). Similarly, human pluripotent stem cells have been shown to overexpress UCP2 and metabolize primarily *via* glycolysis until differentiation, wherein they repress UCP2 expression and switch to primarily oxidative phosphorylation (17). These data suggest that UCP2 may be upregulated in situations where proliferative resources are limited, in situations where cells need to generate metabolites quickly to aid in division, and in states of developmental regression into a more anaplastic phenotype as seen in oncogenesis. The central role of UCP2 in driving the metabolic switch in aggressive neoplasms, and its potential therapeutic implications have yet to be thoroughly investigated. Here we sought to investigate the potential role UCP2 could serve as a biomarker to stratify glioma patients for adjunctive metabolic therapies as well as review the implications of prolonged ROS elevation leading to UCP2 overexpression in malignancy.

## Normal Function of Uncoupling Protein 2

Three homologous mitochondrial uncoupling protein domains exist at locus 11q13.4. These three isoforms, UCP1, UCP2, and UCP3 all pertain to a family of mitochondrial anion carrier proteins. Although they are differentially expressed in different tissue types, they all function to diminish the proton gradient across the inner mitochondrial membrane in mammalian cells by releasing energy in the form of heat rather than by ATP anabolism. Whereas UCP1 is predominantly expressed in brown adipose tissue and facilitates thermogenesis, UCP2 and UCP3 expression is greatest in skeletal muscle and is thought to be more involved in protecting against the cytotoxic effects of ROS (18, 19). UCP2 is located on the inner mitochondrial membrane (IMM) and acts to uncouple the proton gradient across this membrane, of which the primary function is to drive ATP synthesis *via* ATP synthase. Under normal circumstances, protons accumulate in the inner membrane space and ATP synthase facilitates them to readily flow into the matrix, generating ATP in the process. When UCP2 is upregulated, the opposite occurs, in that the exit of anions and protons from the matrix is facilitated. Although the mechanism by which UCP2 facilitates this proton transport is not fully understood, by allowing protons to leak across the IMM, the driving force behind ATP production *via* the electron transport chain is decreased, resulting in heat-energy release.

As is seen in many cancers, this metabolic shift away from mitochondrial cellular respiration causes cells to increasingly depend on glycolysis to meet their metabolic demands. Additionally, recent studies have suggested that UCP2 may have a role in global homeostatic glucose regulation due to its expression in the arcuate nucleus and pro-opiomelanocortin neurons which project into the hypothalamus (14, 20, 21). Consistent with its potential homeostatic role in metabolic function, UCP2 over-expression has been linked to both α and ß-cell dysfunction and increased mRNA transcripts for UCP2 have been detected in the pancreatic islets of several animal models with type 2 diabetes (22–24). A common UCP2 promoter polymorphism -866G/A has been shown to increase transcriptional activity by allowing for easier binding of the pancreatic transcription factor PAX6, increasing the risk of glucose dysregulation and type II diabetes in several human populations (25–29).

## Understood Role of Uncoupling Protein 2 in Gliomas and Other Malignancies

Several cancers, including gliomas, have been observed to upregulate UCP2 expression when compared with their non-neoplastic cells of origin. Upregulation of this protein has been shown to directly increase AKT pathway signaling and enhance glycolysis by activating phosphofructokinase 2, a key regulatory protein in the glycolytic pathway (30). Recent studies suggest that UCP2 plays a critical role in protecting the cell from metabolically generated reactive oxygen species (ROS), which are known to become increasingly present as cells develop more malignant phenotypes. Neoplastic cells are therefore engaged in a cytotoxic positive feedback loop in which they increase carbohydrate metabolism, dramatically increasing intracellular ROS, leading to the upregulation of UCP2, which further dysregulates glycolytic function allowing cells to continue taking in glucose even in states of “satiety” (31, 32).

This phenomenon in which cells shunt metabolic away from oxidative phosphorylation to protect themselves from free radical damage induced by ROS illustrates one possible reason why cancers exhibit high glycolytic dependence even when oxygen is available. UCP2 has also been shown to facilitate mitochondrial Ca^2+^ sequestration from the endoplasmic reticulum specifically (33). While the link between high intracellular Ca^2+^ and apoptosis has long been understood, recent work posits that multiple potentially apoptogenic Ca^2+^ influx pathways exist, due to entering from the extracellular matrix or release from the endoplasmic reticulum (34–37). Therefore, the dramatic increase in UCP2 expression seen across multiple malignancies may also be, in part, due to aiding in sequestering rising intracellular Ca^2+^ in the mitochondria to protect the cell against apoptosis.

**Figure 1:**
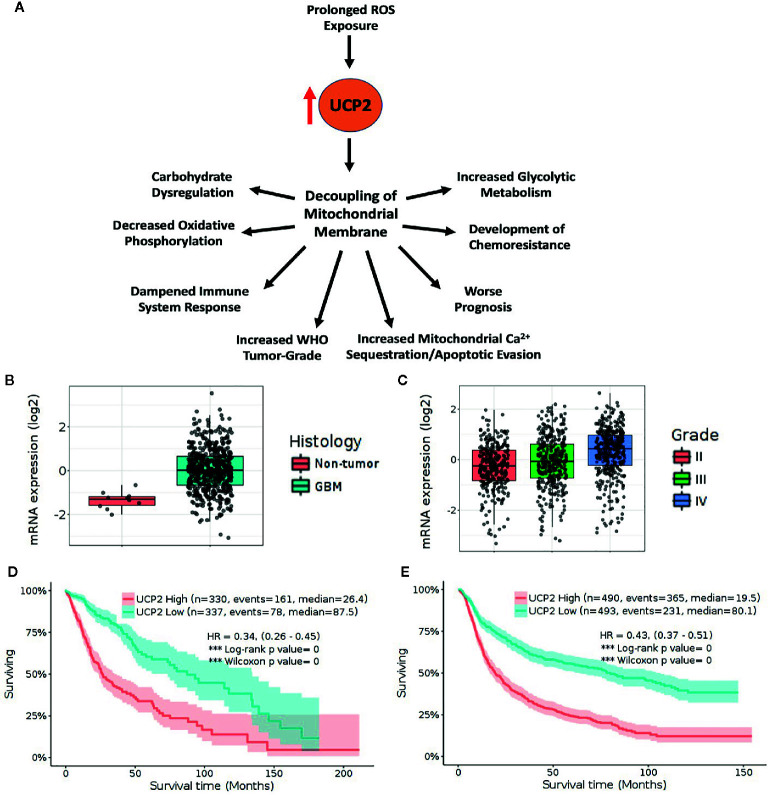
High UCP2 expression is indicative of advanced tumor-grade and is associated with decreased survival. **(A)** UCP2 upregulation has many downstream consequences including worse prognosis. **(B)** TCGA_GBM database platform HG-U133A containing 10 non-tumor and 528 GBM tumor samples shows UCP2 is upregulated in tumor *vs.* non-tumor (*p=* 1.3E-05). **(C)** UCP2 expression correlates with tumor grade as seen in query of CGGA database including 625 low-grade and 388 high-grade gliomas (Grade II *vs.* Grade III *p*= 4.2E-02, Grade II *vs.* Grade IV. *p*= 2.4E-13, Grade III *vs.* Grade IV *p*= 6.3E-07). **(D)** Elevated UCP2 expression is indicative of worse prognosis in survival data from the TCGA GBMLGG dataset containing 515 low-grade and 152 high-grade tumors. **(E)** Elevated UCP2 expression is indicative of worse prognosis in survival data from the CGGA dataset. (Expression data were analyzed *via* pairwise group comparison using *p*-value with Bonferroni correction. Kaplan–Meyer data were analyzed *via* computation of Log-rank *p*-values).

By facilitating apoptotic evasion, overexpression of UCP2 may similarly aide in chemotherapeutic resistance. Overexpression of UCP2 in multiple human cancer cell lines has consistently shown to favor a highly glycolytic phenotype, inhibits ROS accumulation, and prevents apoptosis after exposure to chemotherapeutic agents (38–40). Temozolomide is the chemotherapeutic drug of choice in glioma management and has been shown to trigger dramatic bursts of ROS leading to potential autophagy secondary to ERK activation (41). Previously, our lab has shown that GSCs are resistant to Temozolomide doses well above the peak therapeutic doses reportedly achieved in patient brain tissue and cerebrospinal fluid (42, 43). This resistance may be, in part, due to an increase of UCP2 to protect against ROS accumulation. Taken together, these findings suggest that the upregulation of UCP2 as a cytoprotective mechanism may be largely responsible for inducing this metabolic switch towards aerobic glycolysis, rather than being a consequence of an upstream metabolic alteration (**Figure 1A**).

## Uncoupling Protein 2 as a Potential Biomarker in Glioma

Rising levels of cytotoxic ROS have been shown to directly correlate both with increased glioma grade and with UCP2 expression. We sought to analyze if UCP2 expression alone would correlate with tumor grade. Genomic data on GBM patients from TCGA were analyzed using an open-access brain tumor database, GlioVis (gliovis. bioinfo.cnio.es) (44). Consistent with previous findings, UCP2 expression was shown to be significantly greater in GBM tissue than non-tumor tissue in an analysis of the TCGA GBM database containing 10 non-tumor samples and 528 Glioblastoma samples (*p*<0.05, **Figure 1B**). *UCP2 expression was shown to positively correlate with tumor grade* in both the CGGA RNA-seq database containing 625 low-grade glioma samples and 388 high-grade glioma samples and the TCGA GBMLGG database containing 515 low grade and 152 high-grade glioma samples (*p*<0.05, **Figure 1C**).

More notably, in an analysis of Kaplan-Meyer curves based on gene expression the TCGA GBMLGG shows that, across low- and high-grade gliomas, *higher UCP2 expression is associated with significantly shorter median survival when compared to low UCP2 expression* (High=26.4, Low=87.5 months, **Figure 1D**). Additionally, in a combined analysis of samples from oligodendrogliomas, oligoastrocytomas, astrocytomas, anaplastic oligodendrogliomas, anaplastic oligoastrocytomas, anaplastic astrocytomas, and GBMs the CGGA shows a significant and dramatically poorer median prognosis in tumors which have high UCP2 expression (High=19.5, Low=80.1 months, **Figure 1E**). In short, UCP2 expression increases with increased WHO tumor-grade and is associated with much poorer prognosis across a cohort of brain tumors.

Additionally, high UCP2 expression is known to favor a highly glycolytic metabolic profile, therefore suggesting that more aggressive gliomas may have an increased dependency on glycolysis and may be more susceptible to anti-glycolytic treatments. This finding has large implications for metabolic management of high-grade gliomas. Liquid biopsy has recently been proposed as a method of monitoring or diagnosing tumors *via* non-invasive, low-cost methodology by detecting circulating neoplastic cells, DNA, RNA, or proteins secreted by tumor cells (45). The feasibility of measuring UCP2 levels in patient serum has been previously demonstrated (46–48). Theoretically, patients may be able to establish baseline UCP2 measurements after surgical resection and monitor UCP2 trending upward, indicating disease progression, or trending downward, indicating efficacy of treatment due to either cell death or induction of differentiation. Similarly, following UCP2 levels may aide in the surveillance of low-grade gliomas progressing into more aggressive phenotypes. While UCP2 protein levels have been shown to decrease *via* western blot in response to cellular differentiation, the level of mRNA transcripts were shown to remain relatively stable (16). The consistent presence of mRNA is due to an upstream open reading frame in exon 2 of the UCP2 gene coding for ORF1 which has been shown to strongly inhibit the protein’s expression (49). By regulating this factor, cells are able to quickly increase UCP2 expression in response to metabolic stress without having to generate entirely new transcripts (50, 51). Because of this, UCP2 should be investigated at the protein level should clinicians wish to follow it as a tumor marker in the future. Importantly, determining an individual’s intratumoral UCP2 expression level can lead to targeted metabolic therapeutic interventions. Multi-modality treatment plans incorporating metabolic therapies such as glycolytic inhibition or exogenous ketone body supplementation may be more seriously considered in application to more advanced disease with more dramatic glycolytic demands.

## Therapeutic Implications of Uncoupling Protein 2 Expression Level in Gliomas

The role inflammation plays in glioma progression has yet to be fully understood. While the link between glucose and inflammation has been well documented in the scientific literature, the effect on the tumor’s microenvironment of the metabolic shift accompanying UCP2 upregulation also warrants further investigation. Several studies have demonstrated that, in UCP2 knockout mice, macrophages mount a higher immune response to pathogens when compared with the UCP2 wild-type macrophage response, potentially due to the increased peroxide and superoxide generation inside mitochondria (52–54).

This finding suggests that UCP2 upregulation may be due to not only increased need for ROS mitigation, but also as a way to dampen the immune response to the tumor, allowing evasion of immune-system recognition. This potential effect of UCP2 on the microenvironment is consistent with previous studies showing that gliomas and GBMs are “immuno-cold”, and are oftentimes not engaged or targeted by patients’ immune systems in immunotherapy clinical trials. Conversely, by inducting this metabolic shift which increases glucose availability in the tumor microenvironment, UCP2 may also have a relationship to pro-inflammatory cascades which favor tumorigenesis and progression. Inflammatory tumor associated macrophages (TAMs) in the microenvironment have been associated with more aggressive malignancies with decreased patient survival (55, 56).

**Figure 2:**
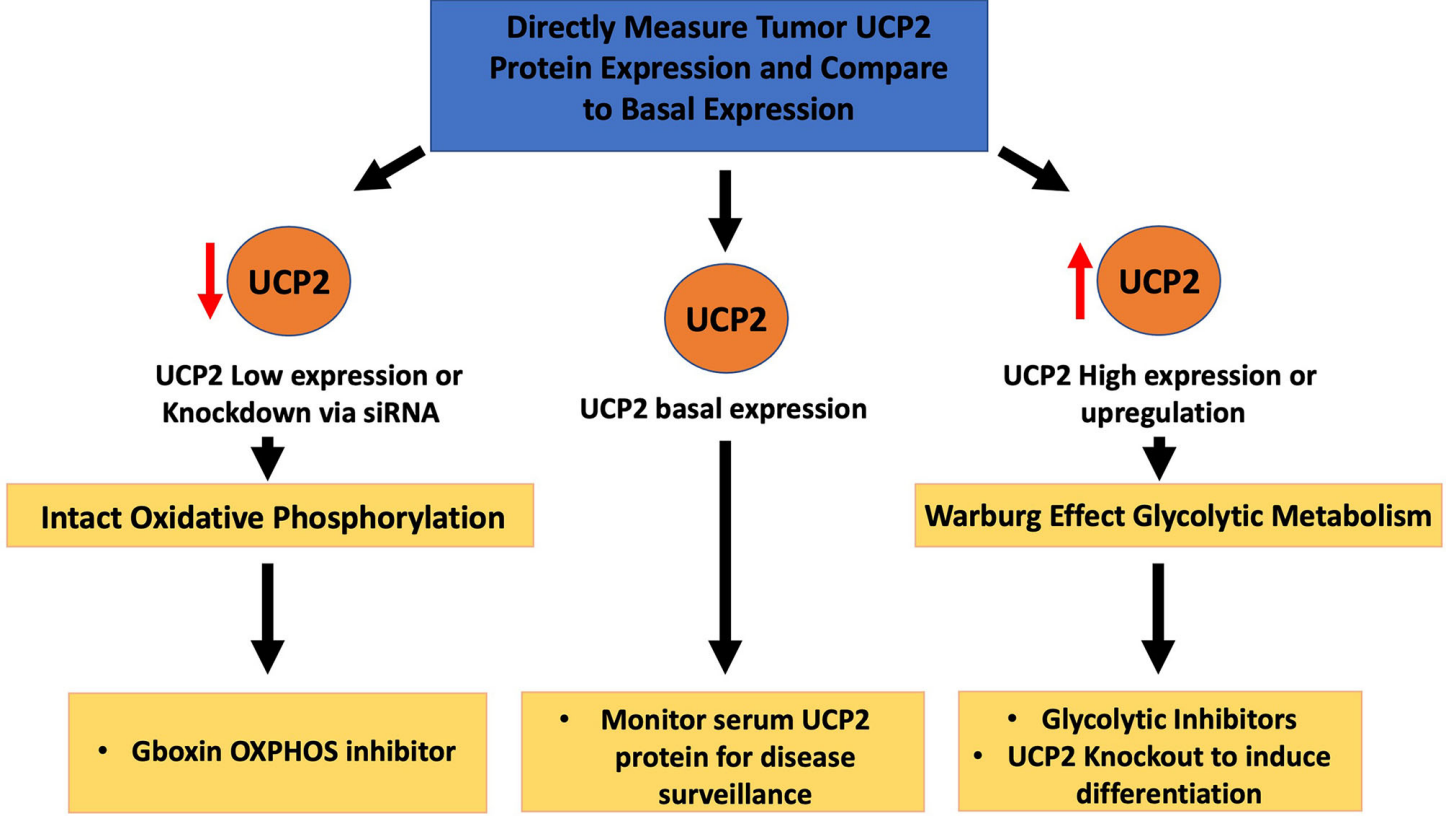
Therapeutic implications of tumor UCP2 expression. UCP2 can be directly measured in tumor tissue and compared to baseline UCP2 expression levels. Tumors with high expression likely to exhibit carbohydrate dysregulation may benefit from adjunctive metabolic treatments such as glycolytic inhibition. Basal levels of UCP2 can be used to monitor tumor progression with potential serum measurements at follow-up. In tumors where UCP2 is inhibited or tumoral expression of UCP2 is very low, mitochondrial metabolism may remain intact suggesting an oxidative phosphorylation inhibitor may be warranted.

Two strategies exist in the metabolic targeting of UCP2 over-expression (**Figure 2**). One strategy is to target tumors with high levels of expression, exhibiting uncoupled oxidative phosphorylation with extremely high levels of glycolytic metabolism, with glycolytic inhibitors or glucose starvation as is suggestive with ketogenic therapy (57). An *in vitro* study on five different GBM cell lines by our lab found that UCP2 was directly upregulated in response to exogenous acetoacetate supplementation, and concurrent glycolytic inhibition produced a dramatic synergistic loss of cell viability (58). Treating UCP2-overexpressing HCT116 cells with 2-deoxy-D-glucose was also shown to halt cell growth, further suggesting the efficacy of glycolytic inhibitor therapy where levels of UCP2 are increased (59). By increasing the glycolytic flux to the tumor, clinicians can aim to lower the supply of glucose available to the tumors while also utilizing anti-glycolytic drugs with minimal toxicity to peripheral tissues.

Another strategy is to inhibit UCP2 directly or preventing its transcription in an effort to slow rampant carbohydrate uptake and restore a more normal metabolic phenotype. In one study, lactate accumulation was diminished after siRNA knockout of UCP2, albeit not to a statistically significantly extent. However, the same study found that a 33% reduction of UCP2 expression resulted in a 22% protection from the proglycolytic-loss of mitochondrial membrane potential, suggesting that UCP2 knockout can restore normal metabolic phenotype *via* enabling oxidative phosphorylation (60). Also, although purine nucleotides are known to inhibit UCP2 expression under normal physiological conditions, the compound genipin successfully inhibited UCP2-mediated proton leak and reversed high-glucose induced ß cell dysfunction in both isolated kidney mitochondria and pancreatic islets (61, 62). Lending to the idea that UCP2 is expressed in highly proliferative, embryonic, stem-like states, UCP2 knockout was shown to suppress murine skin carcinogenesis of both benign papilloma and malignant squamous cell carcinoma (63). Recently, a series of in-vitro experiments demonstrated that UCP2 knockdown inhibited migration, invasiveness, clonogenicity, proliferation, and promoted *via* ROS-mediated cell apoptosis, in addition to reducing tumorgenicity in nude mice by inhibiting the p38 MAPK pathway (64). As previously described, stem cells with high proliferative potential have increased UCP2 expression which is only downregulated upon differentiation. Therefore, knocking out UCP2 may be a promising strategy to induce differentiation and halt cell-division or conversely facilitate apoptosis *via* ROS accumulation.

In regard to intratumoral heterogeneity, some subpopulations of glioblastoma cells may actually exhibit decreased glycolysis, underscoring the need for multi-modal treatment approaches and regular monitoring of the tumor’s metabolic profile to adjust therapy accordingly. One proposed escape mechanism by which cancer cells may evade glycolytic therapy is *via* the p53-mediated induction of SCO2, pushing for oxidative phosphorylation, and TIGAR, which lowers the levels of glycolytic substrate fructose-2,6-bisphosphate (65–67). The mitochondrial permeability transition pore (mPTP) mitigates ROS accumulation *via* transient opening, stabilizing the mitochondrial membrane potential (68). Shi et al. recently demonstrated that glioblastoma and other cancers lack properly functioning mPTP, and that treating these cells with a metabolically stable analogue of Gboxin, an inhibitor of oxidative phosphorylation, inhibits glioblastoma allograft and patient-derived xenografts (69). Therefore, monitoring the expression of UCP2 can help clinicians understand the metabolic profile associated with each unique tumor and give insight as to whether certain metabolic therapies may be effective. Highly glycolytic tumors expressing high levels of UCP2 may benefit from glycolytic inhibition synergizing with glucose deprivation, whereas tumors with low UCP2 expression or with UCP2 knockout metabolizing primarily *via* oxidative phosphorylation may benefit from differentiation and OXPHOS inhibition with Gboxin-like compounds. Patients are in desperate need of novel approaches to combat these malignancies, glioblastoma in particular. UCP2’s implications on tumor metabolism warrants more investigation. Just as PET scans are commonplace in oncologic medicine, further understanding of glioma metabolism may allow full clinical exploitation of these aberrant pathways by implementing targeted therapies into future multi-modal treatment plans.

## Conclusion

Although it has been common scientific knowledge that cancers and gliomas specifically are highly glycolytic, the clinical utility of this tendency has yet to be fully exploited. As ROS increases and gliomas advance in grade, so too does UCP2 expression rise and the tumor become more dependent on glycolytic metabolism. Additionally, high UCP2 expression has shown to correlate with poorer survival outcomes. This finding suggests that more aggressive tumors with high levels of UCP2 expression, which are highly dependent on glycolysis, may benefit from multi-modal treatment approaches which aim to shut down glycolysis. Conversely, UCP2 knockout may aide in restoring normal metabolic phenotype and pushing stem-like cancer cells towards differentiation. UCP2 expression could potentially serve as a biomarker to stratify patients for adjunctive anti-tumor metabolic therapies, particularly in adult and pediatric gliomas.

## Future Perspective

Clinicians in the future may be able to harness UCP2 expression profiles to better direct targeted treatments against aberrant tumor metabolism. Further investigation of UCP2’s role in metabolic reprogramming, the ability to monitor it’s expression as a serum tumor marker, and *in vivo* experiments exhibiting a survival benefit with appropriate stratification for additional therapies is warranted to fully appreciate it’s clinical translatability and utility.

## Author Contributions

FV wrote the manuscript, developed the ideas, conducted the review of literature, and made the figures. SV reviewed the manuscript and helped with the editing process, and helped in guiding FV. RG wrote the manuscript, developed the ideas, and guided FV in creating the figures and focusing on the topic idea. All authors contributed to the article and approved the submitted version.

## Conflict of Interest

The authors declare that the research was conducted in the absence of any commercial or financial relationships that could be construed as a potential conflict of interest.
